# Extracorporeal Membrane Oxygenation for Pandemic (H1N1) 2009

**DOI:** 10.3201/eid1512.091434

**Published:** 2009-12

**Authors:** Michael S. Firstenberg, Danielle Blais, Louis B. Louis, Kurt B. Stevenson, Benjamin Sun, Julie E. Mangino

**Affiliations:** Ohio State University Medical Center, Columbus, Ohio, USA

**Keywords:** Extracorporeal membrane oxygenation, pandemic (H1N1) 2009, acute cardiogenic shock, influenza, viruses, letter, expedited, *Suggested citation for this article:* Firstenberg MS, Blais D, Louis LB, Stevenson KB, Sun B, Mangino JE. Extracorporeal membrane oxygenation for pandemic (H1N1) 2009 [letter]. Emerg Infect Dis [serial on the Internet]. 2009 Dec [*date cited*]. Available from http://www.cdc.gov/EID/content/15/12/2059.htm

**To the Editor**: As the world struggles with the challenges of influenza A pandemic (H1N1) 2009, it is clear that treatment options for critically ill infected patients are suboptimal because deaths continue to be reported in otherwise young and healthy patients. Extracorporeal membrane oxygenation (ECMO) is an established therapeutic option for patients with medically refractory cardiogenic or respiratory failure. We describe the successful use of ECMO in a patient with complicated pneumonia and influenza A pandemic (H1N1) 2009 virus infection.

Our patient, a 21-year-old woman who was 4 months postpartum, had poorly controlled insulin-dependent diabetes (hemoglobin A1C level 13.2 mg/dL). She sought treatment at another hospital after 3 days of respiratory symptoms, a productive cough after working in her garden, and a fever >103°F. Her condition rapidly deteriorated, and she required mechanical ventilation, vasoactive medications, and drotecogin-α (Xigris; Eli Lilly and Company, Indianapolis, IN, USA) for profound shock.

The patient was then transferred to Ohio State University Medical Center on August 24, 2009; at admission she exhibited hypotension (83/43 mm Hg) and tachycardia (159 bpm), despite having received high doses of vasoactive medications (norepinephrine 1.0 µg/kg/min, phenylephrine 2.0 µg/kg/min). A transthoracic echocardiograph showed severe biventricular failure (ejection fraction 5%–10%); peak tropinin level was 6 mg/dL. Arterial blood gas confirmed metabolic acidosis (pH 7.12, partial carbon dioxide pressure [pCO_2_] 48 mm Hg, pO_2_ 117 mm Hg, HCO_3_ 15.3 mmol/L). Despite fluid resuscitation and administration of epinephrine (0.06 µg/kg/min), her condition failed to improve, and she was given femoral vein–femoral artery ECMO.

A comprehensive search for infectious causes was undertaken. Treatment with broad-spectrum empiric antimicrobial drugs such as linezolid (Pfizer, Inc, New York, NY, USA), piperacillin/tazobactam (Wyeth, Madison, NJ, USA), and doxycycline (Pfizer, Inc) and the antiviral drug oseltamivir (Tamiflu; Roche Laboratories Inc., Nutley, NJ, USA), 150 mg 2×/d, was started. Respiratory cultures were positive for methicillin-sensitive *Staphylococcus aureus* and *Aspergillus glaucus.* Nafcillin and voriconazole were added to the treatment regimen. PCR of a bronchoalveolar lavage specimen later identified pandemic (H1N1) 2009 virus. The patient was weaned from ECMO on hospital day (HD) 10 and extubated on HD11. Repeat cardiovascular evaluation showed normal biventricular function and no coronary disease. She was discharged from hospital for rehabilitation on September 15, 2009 (HD 22), with an oxygen saturation of 98% on room air and is now fully recovered.

The use of ECMO is an established option for patients with medically refractory acute and reversible cardiopulmonary failure (Table) (*1**–**3*). For isolated respiratory failure, veno–veno support can be used by femoral vein to femoral vein or femoral vein to right internal jugular vein cannulation. With concomitant cardiogenic shock, veno–arterial cannulation may be required with cannulation of the right internal jugular or femoral vein for outflow, and for inflow, the femoral artery directly or the axillary artery by a surgically placed side graft. Central venous (right atrium) and arterial (ascending aorta) cannulation is an option but requires median sternotomy.

**Table:** Relative indications and contraindications for extracorporeal membrane oxygenation*

Characteristics
Cardiac support (*1*)
Cardiac index <2.2 L/min/m^2^
Systolic pressure <90 mm Hg
Pulmonary capillary wedge pressure >20 mm Hg
Central venous pressure >20 mm Hg
Two high-dose inotropic medications (Including intraaortic counter pulsation)
Respiratory support (*2*)
Murray score >3.0 based on:
PaO_2_/FiO_2_ ratio
No. infiltrated quadrants on chest radiograph
Positive end-expiratory pressure requirement
Pulmonary compliance
Uncompensated hypercapnea (pH<7.2)
Contraindications
Advancing age (>70 y)
Prolonged mechanical ventilation (>7 d)
Surgically correctable causes
Pneumothorax, effusions, endoluminal obstructions
Intracardiac shunts, valvular pathology, incomplete revascularization
Medical problems incompatible with prolonged survival
Advanced malignancies
Contraindications to anticoagulation
Irreversible neurologic dysfunction (dementia, stroke, hemorrhage)
Medical futility (i.e., prolonged CPR, multiorgan failure)

*CPR, cardiopulmonary resuscitation; PaO_2_, partial pressure of oxygen in arterial blood; F_1_O_2_, concentration of inspired oxygen.

This case is not the first reported use of ECMO for respiratory failure secondary to viral pneumonia (*4*), and recently, ECMO was used with limited success for complications of pandemic (H1N1) 2009 (*5*). Its broader use in treating critically ill patients has been limited, however, because ECMO requires substantial institutional and multidisciplinary commitment for implementation and is typically only available at major medical centers offering cardiovascular surgery.

Although we cannot say specifically why our patient survived, clearly, aggressive and comprehensive empiric treatment, physiologic support, and close multidisciplinary communication were vital to managing the condition of this critically ill patient. ECMO may have assisted in organ recovery and patient survival. However, further studies should be conducted to critically evaluate ECMO in the armamentarium of therapeutic options for severe pandemic (H1N1) 2009 respiratory failure.
